# Metavalent or Hypervalent Bonding: Is There a Chance for Reconciliation?

**DOI:** 10.1002/advs.202308578

**Published:** 2023-12-07

**Authors:** Matthias Wuttig, Carl‐Friedrich Schön, Dasol Kim, Pavlo Golub, Carlo Gatti, Jean‐Yves Raty, Bart J. Kooi, Ángel Martín Pendás, Raagya Arora, Umesh Waghmare

**Affiliations:** ^1^ I. Institute of Physics Physics of Novel Materials RWTH Aachen University 52056 Aachen Germany; ^2^ Jülich‐Aachen Research Alliance (JARA FIT and JARA HPC) RWTH Aachen University 52056 Aachen Germany; ^3^ Green IT (PGI 10) Forschungszentrum Jülich GmbH 52428 Jülich Germany; ^4^ Department of Theoretical Chemistry J. Heyrovský Institute of Physical Chemistry Dolejškova 2155/3 Prague 18223 Czech Republic; ^5^ CNR‐SCITEC Istituto di Scienze e Tecnologie Chimiche “Giulio Natta” sezione di via Golgi, via Golgi 19 Milano 20133 Italy; ^6^ CESAM B5 Université de Liège Sart‐Tilman Liège B4000 Belgium; ^7^ Zernike Institute for Advanced Materials University of Groningen Nijenborgh 4 Groningen 9747AG The Netherlands; ^8^ Departamento de Química Física y Analítica Julián Clavería 8 Oviedo 33006 Spain; ^9^ Theoretical Sciences Unit School of Advanced Materials JNCASR Jakkur Bangalore 560064 India

**Keywords:** hypervalent bonding, material design, material maps, metavalent bonding, quantum chemical bonding descriptors

## Abstract

A family of solids including crystalline phase change materials such as GeTe and Sb_2_Te_3_, topological insulators like Bi_2_Se_3,_ and halide perovskites such as CsPbI_3_ possesses an unconventional property portfolio that seems incompatible with ionic, metallic, or covalent bonding. Instead, evidence is found for a bonding mechanism characterized by half‐filled p‐bands and a competition between electron localization and delocalization. Different bonding concepts have recently been suggested based on quantum chemical bonding descriptors which either define the bonds in these solids as electron‐deficient (metavalent) or electron‐rich (hypervalent). This disagreement raises concerns about the accuracy of quantum–chemical bonding descriptors is showed. Here independent of the approach chosen, electron‐deficient bonds govern the materials mentioned above is showed. A detailed analysis of bonding in electron‐rich XeF_2_ and electron‐deficient GeTe shows that in both cases p‐electrons govern bonding, while s‐electrons only play a minor role. Yet, the properties of the electron‐deficient crystals are very different from molecular crystals of electron‐rich XeF_2_ or electron‐deficient B_2_H_6_. The unique properties of phase change materials and related solids can be attributed to an extended system of half‐filled bonds, providing further arguments as to why a distinct nomenclature such as metavalent bonding is adequate and appropriate for these solids.

## Graphical abstract

Recently, the bonding in crystalline phase change materials has been disputed, with two opposing views emerging. The bonds are either described as electron‐deficient or electron‐rich. Here, a coherent view of bonding is presented comparing density‐ and orbital‐based quantum‐chemical techniques. Consistently, both describe phase change materials as electron‐deficient, i.e. metavalent solids with a remarkable and well‐defined set of properties.

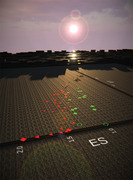

Understanding and designing material properties has been one of the prime goals of material science. Interestingly, a number of inorganic solids, including chalcogenides, pnictogens, and halide perovskites are characterized by a rather unusual property portfolio, challenging our understanding of materials. These solids show strong optical absorption, suitable for photovoltaic applications and optical data storage. Yet, they feature soft and anharmonic bonds, small effective masses,^[^ ^1^ ^]^ as well as pronounced levels of static and dynamic disorder, even leading to disorder‐induced localization.^[^ ^2^ ^]^ Surprisingly, this property portfolio characterizes a range of different material families including monochalcogenides like GeTe, SnTe, PbTe, and PbSe, pnictogens like Sb and Bi, as well as sesquichalcogenides such as Sb_2_Te_3_ or Bi_2_Se_3_. Ternary chalcogenides like AgSbTe_2_ and even halide perovskites like CsSnI_3_, CsPbI_3_, or MAPI (CH_3_NH_3_PbI_3_) also possess this unusual property combination.^[^ ^3^ ^]^

Early on, scientists have been pondering on the origin of these properties. Lucovsky and White suggested to call the interatomic bonding in monochalcogenides such as GeTe resonant bonding.^[^ ^4^ ^]^ They compared the situation with benzene, the famous textbook example of resonant bonding devised by Pauling more than 60 years ago.^[^ ^5^ ^]^ Later on, it was shown that many crystalline phase change materials including Ge_2_Sb_2_Te_5_ or AIST (Ag and In doped Sb_2_Te) possess similar properties as GeTe. Hence, it was proposed to employ the concept of resonant bonding to explain and design the properties of crystalline phase change materials.^[^ ^6^ ^]^ A few years later, it became clear that applying this concept to explain the characteristics of phase change materials runs into difficulties. Solids like graphite, graphene, or carbon nanotubes, which have an electronic configuration that resembles the situation in benzene, have properties that differ significantly from the chalcogenides and halide perovskites discussed here.^[^ ^7^ ^]^ Hence, it is questionable if the same bonding mechanism is at play. This concern was corroborated when it was found that the bond rupture in laser‐assisted field evaporation is unusual for the chalcogenides discussed here.^[^ ^8^ ^]^ Crystalline chalcogenides like GeTe or Bi_2_Se_3_ show a high probability of multiple events, i.e., the formation of several ions upon bond rupture. This is not found for solids that employ ionic, covalent, or metallic bonding.^[^ ^9^ ^]^ Carbon nanotubes, which can be described as resonantly bonded, also do not show the unconventional bond rupture of crystalline chalcogenides.^[^ ^10^ ^]^ These findings support the view that the unique properties of these chalcogenides and related compounds justify giving the underlying bonding mechanism a name, which differentiates it from ionic, metallic, and covalent bonding, as well as resonant bonding as in graphite, graphene, and benzene. The name “metavalent bonding” has been suggested to stress the finding that these compounds are beyond ordinary covalent, i.e., two centers–two‐electron (2c–2e) bonding,^[^ ^7^ ^]^ yet also differ from ionic and metallic bonding. Metavalent solids are located in a special region of a map, spanned by the number of electrons transferred and electrons shared between adjacent atoms.^[^ ^11^ ^]^ As displayed in **Figure** **1**, these compounds typically share ≈1 electron (half of an electron pair) between neighboring atoms and utilize a rather small charge transfer, intermittent between covalent and metallic bonding. Nevertheless, the properties of these compounds are distinctly different from both metals and covalently bonded solids.

**Figure 1:**
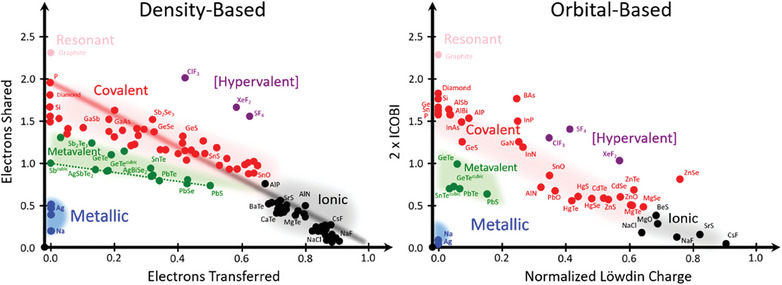
2D maps classifying chemical bonding in solids. The map on the left is obtained from a density‐based approach, while the map on the right is determined from an orbital‐based approach. The *x*‐ and *y*‐axis are spanned by the number of electrons shared between adjacent atoms and the electron transfer renormalized by the formal oxidation state (density‐based calculation), or twice the bond order integrated crystal orbital bond index (ICOBI) as well as the Löwdin charge renormalized by the formal oxidation state (orbital‐based calculation), respectively. Different colors characterize different material properties and have been related to different types of bonds.^[^ ^7^, ^22^ ^]^ With the two different approaches, classes of solids (covalent, ionic, metallic, and metavalent) are located in similar locations in both maps. This indicates that both approaches provide a consistent description of chemical bonds. Electron‐rich compounds, such as XeF_2_ or SF_4_, frequently considered to be hypervalent, and metavalent solids, like GeTe or Sb_2_Te_3_, are located in different regions of the map. This is indicative of significant differences in their electronic states in the vicinity of the Fermi level, which governs bonding. The dashed green line in the density‐based calculations identifies the location of solids with perfect octahedral arrangements like cubic Sb, AgSbTe_2,_ and PbS.

## 1 Do Density‐ and Orbital‐Based Techniques Provide a Coherent View on Bonding in Solids?

Recently, Jones, Elliott, and Dronskowski^[^ ^12^ ^]^ (JED) have questioned the idea that crystalline phase change materials like GeTe, or Sb_2_Te_3_, topological insulators such as Bi_2_Se_3_ or halide perovskites like CsPbI_3_ employ metavalent bonding. They express the concern that a density‐based approach to characterize chemical bonding as we used in^[^ ^11^, ^13^ ^]^ could be inappropriate and rather advocate the usage of an orbital‐based approach.

Fortunately, the concern that a density‐based approach is misleading can be refuted. Recent developments in quantum chemistry enable the characterization of bonds between atoms in solids both within orbital‐based and density‐based approaches.^[^ ^14^, ^15^, ^16^ ^]^ Recently Dronskowski and coworkers have developed an orbital‐based code, which is publicly available,^[^ ^17^ ^]^ enabling a direct comparison with the bonding descriptors derived from our density‐based calculations. The results obtained by such analyses are shown in Figure 1. On the left‐hand side, the results of a density‐based approach are depicted. Here, two quantum chemical bonding descriptors are employed to characterize bonding. The *x*‐axis is spanned by the number of electrons transferred between neighboring atoms (normalized by the oxidation state), while the *y*‐axis is the number of electrons shared (twice the number of electron pairs formed) between adjacent atoms.^[^ ^11^ ^]^ On the right‐hand side, the results of an orbital‐based analysis of chemical bonding are displayed, according to Müller et al.^[^ ^17^ ^]^ Here, the axes is spanned by two properties closely related to the properties on the left. As the *x*‐axis, the Löwdin charge (normalized by the formal oxidation state) is employed as a measure of the electron transfer. This quantity in the present case is similar to the definition of electron transfer in the density‐based method, since (for binary systems) the Löwdin charges of the bonded atoms are opposite in sign but equal in magnitude. The *y*‐axis is spanned by the ICOBI (integrated crystal orbital bond index),^[^ ^17^ ^]^ a generalization of the bond index according to Wiberg and Mayer,^[^ ^18^, ^19^, ^20^ ^]^ as a measure of half the number of electrons shared, i.e., the bond order between adjacent atoms. Indeed, the Wiberg–Mayer bond order is the orbital‐space analog to half the number of electrons shared between adjacent atoms (ES).^[^ ^21^ ^]^ It is reassuring to note that both calculation schemes produce very similar maps, i.e., they reveal similar regions for the different bonding mechanisms. Ionic bonding, as expected, is identified by pronounced charge transfer but rather limited electron sharing, and is thus located in the lower right corner. Covalent bonding, on the contrary, is dominated by electron sharing, with the formation of an electron pair, i.e., a bond order of ≈1, as exemplified by diamond. Such solids are hence found in the upper left corner. Metals are electron‐deficient since they do not have enough electrons to form ordinary covalent bonds with all nearest neighbors and are thus located in the lower left corner. Hence the findings of density‐based and orbital‐based analysis of chemical bonding are rather consistent. Only for a very small number of materials, noticeable differences in the quantum chemical bonding descriptors can be seen, such as the small Löwdin charges found for AlN or the small bond order for CdTe. These differences found for a small number of materials can help to better understand the pros and cons of the different approaches. At the same time, for the vast majority of solids, there is rather good agreement for the number of electrons shared (twice the Mayer bond order) for most compounds. More importantly, all compounds that we have identified as metavalent are located in the same region for both maps. Crystalline phase change materials like GeTe or Sb_2_Te_3_ and PbTe are located in a distinct region between metallic and covalent solids. Both computation schemes identify crystalline phase change materials as having small charge transfer and sharing of about half an electron pair (bond order of ≈½, i.e., sharing ≈1 electron) between neighboring atoms. The similarity of both maps offers great hope that such bonding analyses can help to cease the decade‐old battles between orbital‐ and density‐based approaches to chemical bonding and enable a period of due reconciliation. While this is clearly good news for everyone who wants to analyze bonds in solids, it is possibly not entirely surprising, since the orbital‐based and the density‐based analyses used here are applied to the same computed wavefunctions.

## 2 Are Crystalline Phase Change Materials Electron‐Deficient or Electron‐Rich?

Given the good agreement between orbital‐ and density‐based approaches, one can wonder how conflicting views on bonding can still exist. The remaining dispute we are trying to settle is, whether the solids in the green region of the map are electron‐deficient (metavalent), or electron‐rich (hypervalent). JED postulates the bonding in crystalline phase change materials to be hypervalent multi‐center (3c–4e) bonding, closely resembling the bonding in XeF_2_.^[^ ^12^ ^]^ This is surprising since metavalently bonded solids are located in Figure 1 for both the density‐based and the orbital‐based calculations in a region of the map, which is distinctively different from that of the hypervalent compounds XeF_2_, ClF_3_, and SF_4_. While metavalent solids are located between covalent and metallic compounds, hypervalent compounds are located in a region best described as iono‐covalent (polar covalent). Hence a visual inspection of Figure 1 already casts some doubt on the statement that the bonding in metavalent solids resembles the bonding in electron‐rich XeF_2_. Nevertheless, we want to confirm or refute, if the electronic bonding configuration in XeF_2_ indeed closely resembles the one in crystalline phase change materials like GeTe. In doing so, we follow a strategy outlined in Roald Hoffmann's compelling paper “How Chemistry and Physics Meet in the Solid State.”^[^ ^23^ ^]^

**Figure 2:**
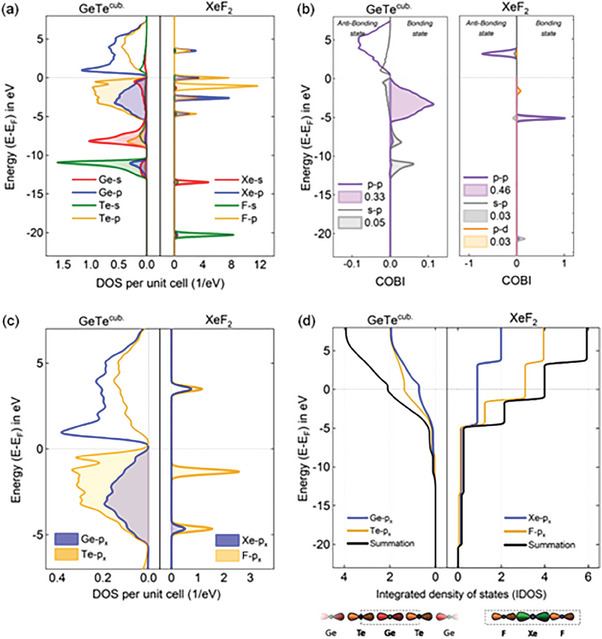
Density of States (DoS) a), Crystal Orbital Bond Index (COBI) b), DoS of the p‐electrons for the *x*‐direction c) and integrated DoS for the *x*‐direction d) for cubic GeTe and XeF_2_. In both cases, the states between the Fermi level (E_F_), and up to ≈−5 eV below E_F_, which govern bonding, are dominated by p‐states, while the contribution of s‐states is very small. The COBI confirms that the bonding in GeTe is governed by p–p orbital overlap, with a small contribution from s to p orbital overlap. This is similar in XeF_2_, where there is also only a small contribution from s to p orbital overlap. Two differences can be noted between XeF_2_ and GeTe, the p–p orbital overlap is slightly larger for XeF_2_, and there is an additional p–d orbital overlap, which does not exist for GeTe (Please note the different scales for the *x*‐axes). In c), the DoS for the p‐states are only shown for the bond direction (i.e., *x*‐direction for XeF_2_) and one of the three orthogonal bond directions in cubic GeTe. A visual comparison of the fraction of occupied and empty p‐states shows that for GeTe half of the p‐states are shown, while for XeF_2_ 2/3 of the p‐states per atom are occupied. This is confirmed by the integrated DoS for this bond direction, which shows that each GeTe atom pair only contributes one electron to their bond, creating a half‐filled bond.

First, we compare the density of states of the frontier orbitals, i.e., the states/orbitals close to the Fermi level as suggested by.^[^ ^23^ ^]^ In total, there are ten valence electrons in GeTe (2 s‐ and 2 p‐electrons for Ge and 2 s‐ and 4 p‐ electrons for Te) and 22 valence electrons for XeF_2_ (2 s‐ and 6 p‐electrons for Xe and twice 2 s‐ and 5 p‐ electrons for F). To understand their contribution to bonding, the density of states (DoS) is determined. In **Figure** **2**a, the orbital resolved DoS for the solid, i.e., a molecular crystal of XeF_2_ is depicted and compared with cubic GeTe. Interesting similarities and pronounced differences are seen. This figure shows that in XeF_2_, the outermost s‐orbitals of Xe and F have energies far below the Fermi level, with values of 14 and 21 eV, respectively. Hence, they do not contribute significantly to bonding. On the contrary, in the region between E_F_ and −5.5 eV, there are predominantly p‐states and practically no s‐states. This also holds for GeTe, where the Te s‐state is located at ≈−11 eV, while the Ge s‐state is located at ≈−8 eV. As for XeF_2_, also for GeTe, both s‐states are thus far below the Fermi level and hence should not be particularly relevant for bonding. Yet, there is a significant difference in the fraction of the p‐states occupied. While for GeTe half of the p‐states are occupied, a larger fraction is occupied for XeF_2_.

To substantiate and quantify the contribution of different electrons to bonding, analytical tools can be employed. In the following, we utilize the Crystal Orbital Bond Index (COBI), an energy‐ and orbital‐resolved measure of the bond order.^[^ ^17^ ^]^ As shown in the Supporting Information, very similar conclusions can also be obtained from an analysis of the electron pair density (Section S1, Supporting Information). The COBI shows that bonding is dominated by p–p orbital overlap for both GeTe and XeF_2_. In the Supporting Information, we will discuss in detail, why the s‐ electrons hardly contribute to bonding in GeTe (sections S1 and S2, Supporting Information). To analyze the contribution of the p‐electrons, the density of states of these p‐electrons are depicted focusing on the molecular bond direction in XeF_2,_ comparing the results with the DoS of the p‐states in x‐ direction for cubic GeTe. Comparing the DoS in Figure 2c with the COBI in Figure 2b shows that the lowest lying p‐states in XeF_2_ at –5 eV are bonding states, while the highest lying (unoccupied) states at ≈3 eV are antibonding. The occupied states ≈−2 eV are predominantly nonbonding, as already anticipated in the Pimentel model.^[^ ^24^ ^]^ Such nonbonding electrons are *not* present in GeTe. In Figure 2d the integrated DoS for the bond direction of XeF_2_ and one of the three orthogonal bond directions in GeTe is shown. This figure confirms that XeF_2_ utilizes electron‐rich (hypervalent) bonding since there are two electrons available between adjacent atoms, leading to a 3c–4e configuration. For GeTe, on the contrary, there is only one electron available (half of an electron pair) to form bonds between Ge and Te. Hence, GeTe is electron‐deficient. This conclusion is also supported by data obtained from a projection of the electronic wavefunctions of XeF_2_ and GeTe on the corresponding atomic orbitals. **Table** **1** shows the integrated partial density of states for the valence DoS of GeTe and XeF_2_ (from ≈−5 eV to the Fermi energy (E_F_)). We can limit our analysis to that energy range as the states at lower energies have COBI values close to 0. The numbers are obtained using Lobster projections onto atomic orbitals.^[^ ^25^ ^]^ As expected, in XeF_2_, the s contribution is close to 0. For GeTe, the s‐electron contribution is governed by Ge atoms but is also quite small. The Ge s‐orbital only reaches 1/6 of the Ge p‐electron contribution. Instead, the bonding between Ge and Te is governed by the overlap of adjacent p‐orbitals. The number of p electrons contributing to bonding from Ge is almost exactly one‐half of the number of Te p electrons, and close to 2. In total, ≈6 p‐electrons contribute to this energy range. Since each atom has six nearest neighbors in cubic GeTe, there is just a single p‐electron (half of an electron pair) available for bond formation, in good agreement with the orbital and density‐based bonding analyses.

**Table 1:** Integrated partial densities of states for both GeTe and XeF_2_. These numbers correspond to the number of electrons in the uppermost valence DOS block (from ≈−5 eV to the Fermi level, see Figure 2), which provide most of the ICOBI value. Please note that Xe only forms bonds to two adjacent F atoms.

Bonding	s–electrons	p–electrons		s–electrons	p–electrons
Ge	0.30	1.88	Xe	0.09	4.72
Te	0.05	3.72	F	0.04	5.48

There is one additional point that will be briefly discussed before this section is summarized. As seen already from a comparison of the COBI data, in GeTe the bond order for cubic GeTe is below 0.5, while it is slightly above 0.5 for XeF_2_ and even considerably higher for the density‐based calculations in Figure 1 (with a bond order of 0.83 (ES = 1.66). It is reasonable to ponder where this difference between GeTe and XeF_2_ in terms of bond order comes from, and why the orbital‐based calculations show a smaller bond order than the density‐based calculation. For a solid with a bond order of 0.5, we would indeed expect an ICOBI value close to 0.5. In the case of GeTe, there are two contributions that lower the ICOBI. There is some charge transfer between Ge and Te, which reduces the electrons available to overlap. This is seen in Figure 1a, where the data for cubic metavalent solids fall on a slanted line, where ES decreases with increasing ET. Furthermore, there is also a small antibonding contribution from the overlap of Ge s‐ and Te p‐states (discussed in detail in the supplement), due to the opposite dispersion of s‐ and p‐states. Hence, it is very plausible that GeTe has a bond order slightly smaller than 0.5. This is not the case for XeF_2_. The ICOBI already provides part of the explanation for this difference between XeF_2_ and GeTe. It reveals that there is a contribution between F p‐states and Xe 4d‐states, not considered in the Pimentel model. If this contribution is not considered, the ICOBI is 0.99. If the 4d orbital is considered as well, the ICOBI increases to values slightly above 1 (1.03). However, in the density‐based calculations, an even larger value is observed. This is explained in detail in the supplement, where it is shown that upon bond formation there is even overlap between the F 2p‐state and the Xe $5d_{z^{2}}$ state. The corresponding domain averaged fermi hole (DAFH) orbital is shown in Figure S1 (Supporting Information). This Xe $5d_{z^{2}}$ orbital, and any other 5d orbital of Xe for this matter, is ignored in the present orbital‐based calculations. Since this orbital is not included in the bond analysis, it also cannot be projected onto, and hence a smaller ICOBI value results.

In summary, in this section, it is shown that the electronic configuration relevant for bonding, i.e., the electronic states in the vicinity of the Fermi level (shown in Figure 2a) are quite different for XeF_2_ and (cubic) GeTe. This conclusion has been further corroborated by a detailed analysis of the density of states (DoS) in conjunction with an analysis of bonding using orbital‐based quantities. The ICOBI shows that in GeTe about half an electron‐pair forms between adjacent atoms, leading to an effective bond order of 1/2. This bond order is realized predominantly by the p–p σ orbital overlap, with a small contribution from p–p π orbital overlap to (Ge) s – (Te) p σ orbital overlap. Hence, GeTe shows electron‐deficient bonding (2c–1e) bonding. In XeF_2_ on the contrary, the DoS shows in the bond direction 4 electrons between 3 atoms forming σ‐bonds, i.e., a 3c–4e bonding, which is also denoted as hypervalent bonding. Two of these electrons are in predominantly nonbonding states, while two others are in bonding states. We note in passing that the usefulness of the term “hypervalent bonding” has been vividly debated recently.^[^ ^26^ ^]^ There also have been long‐standing debates about the adequacy of Pimentel's model.^[^ ^27^ ^]^ Indeed, the bond analysis presented above shows that Pimentel's model does not adequately describe the bonding in XeF_2_. As shown in more detail in the supplement, there is additional orbital overlap, particularly with Xe d‐states, which further increases the bond order in XeF_2_ compared to GeTe. While both debates are interesting and important on their own, in terms of understanding the bonding of crystalline phase change materials it is only important to stress that the bonding in XeF_2_ and GeTe are so different that an identification of bonding in GeTe as electron‐rich multi‐center bonding is unjustified.

## 3 Do Unconventional Material Properties Justify a Separate Name for the Responsible Bonds?

**Figure 3:**
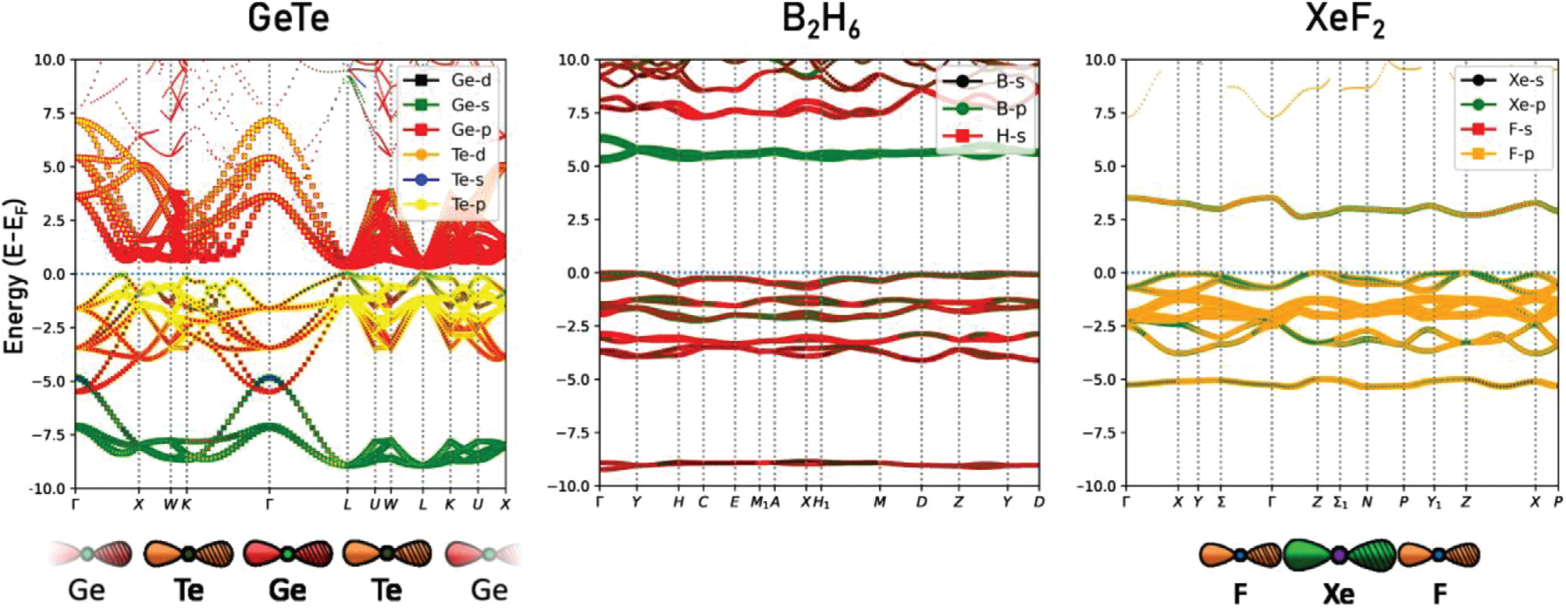
Band structure of three different solids: Electron‐rich and electron‐deficient molecular crystals (XeF_2_ and B_2_H_6_, respectively and electron‐deficient solid (GeTe). Pronounced differences in the band structure are noted. The two molecular crystals have large bandgaps and rather flat bands, characteristic of an insulating state, while the electron‐deficient (metavalent) solid (GeTe) has the characteristic band structure of an incipient metal, due to the existence of an infinite chain of orbitals.

In the last section, it was shown that the electronic configuration, i.e., the occupation of the frontier orbitals in XeF_2_ and GeTe are quite different. While XeF_2_ is electron‐rich, GeTe is electron‐deficient. This immediately raises the question if these differences are also related to significant differences in relevant material properties. After all, one might argue that both solids possess a bond order of ≈½, if characterized by the ICOBI. However, a discussion of the bond order alone is insufficient to describe bonds in solids and molecules, at least a second bonding descriptor, such as ET or the Löwdin charge is needed to distinguish metavalent, hypervalent, and iono‐covalent bonding as shown in Figure 1. We will even see that there is another important difference not captured in Figure 1, which distinguishes metavalent GeTe and hypervalent XeF_2_. To make this point clear and to emphasize the differences between GeTe and XeF_2_, we follow an argument by Hoffmann, who stresses^[^ ^23^ ^]^ that there is a close relationship between the frontier orbitals of a solid and the band structure in the vicinity of the Fermi energy (E_F_). If the bonding in XeF_2_ indeed should be similar to be bonding in GeTe, we would expect to find clear similarities in the band structure. The comparison of these two band structures is shown in **Figure** **3**. Pronounced differences are clearly seen, which is indicative of pronounced differences in bonding between these two solids. XeF_2_ shows a large bandgap of more than 3 eV and rather flat bands. This is fully consistent with the outward appearance of this solid, which forms transparent crystals. Crystalline GeTe instead shows metallic‐like luster, in line with its small bandgap. Furthermore, the strong overlap of adjacent p‐orbitals leads to a pronounced dispersion in the band structure. Representative for crystalline phase change materials like GeTe or thermoelectrics like PbTe is the characteristic avoided crossing of the bands at the L‐point (and also at a point along the ΓK‐direction) derived from overlapping p‐orbitals.

Yet, there are other solids that form electron‐deficient bonds. Almost 70 years ago, Rundle and coworkers already identified a class of molecules that form electron‐deficient bonds.^[^ ^28^ ^]^ A representative of this class is diborane (B_2_H_6_). Diborane also forms molecular crystals. The band structure of diborane (see Figure 3) closely resembles the band structure of XeF_2_. This is no surprise since both materials form molecular crystals, characterized by weak coupling between adjacent molecules and hence weak dispersion. The comparison of these three solids helps to draw another important conclusion. The band structure and hence many crucial properties of phase change materials like GeTe (and many related chalcogenides) are very different from that of XeF_2_, not only because the frontier orbitals are different. There are also pronounced differences since cubic GeTe forms an infinite chain of overlapping p‐orbitals. It is this half‐filled infinite chain of p‐orbitals, which neither exists in XeF_2_ nor in B_2_H_6_, which is responsible for many relevant material properties of crystalline phase change materials. Hence, the uniqueness of GeTe is not the electron deficiency alone, it is also the fact that we have infinite chains of atoms (in all three directions) with overlapping p‐orbitals with half‐filling. Atoms are bonded together predominantly through σ channels of *p–p* interactions. This results in spectacular electron‐hole symmetry: conduction and valence bands are almost symmetric with respect to Fermi energy, E_c_(k)≈‐E_v_(k) at each wave vector $\vec{k}$. A slight deviation from this symmetry of the band structure arises from the weak interaction of *p*‐bands with the lone pair *s*‐bands lower in energy. This property is inherited from the parent metallic state of the cubic group V elemental crystal.^[^ ^29^ ^]^ Hence, GeTe‐like crystals are appropriately termed as incipient metals.

**Figure 4:**
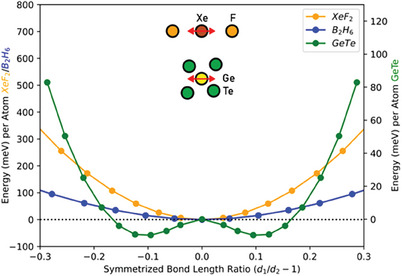
Potential energy curve with respect to Peierl distortion. The two electron‐rich and electron‐deficient molecular crystals (XeF_2_ and B_2_H_6_) show no tendency toward a Peierls distortion, while GeTe lowers its energy through a Peierls distortion, characteristic of the competition between electron delocalization and electron localization.

The bandgap opening upon distortion in monochalcogenides like GeTe describes a situation that Peierls already discussed many decades ago, initially in a one‐dimensional model.^[^ ^30^ ^]^ He argued that half‐filled bands are characterized by electronic instability, which leads to an energy‐lowering distortion. The corresponding atomic rearrangement is now frequently denoted as a Peierls distortion. GeTe also reveals such a Peierls distortion, which is shown in **Figure** **4**, while neither XeF_2_ nor B_2_H_6_ shows this instability.

The differences mentioned above between electron‐deficient GeTe and molecular crystals of electron‐rich XeF_2_ and electron‐deficient B_2_H_6_ are important for several reasons. Electron‐rich and electron‐deficient molecules have been known for more than half a century. They are usually classified as part of the wider family of molecules with covalent bonds since they have no characteristic features that necessitate a distinction from ordinary covalent molecules like H_2_ or CO. Electron‐deficient solids like GeTe or PbTe differ substantially from molecular crystals like XeF_2_ or B_2_H_6_. They also have properties that are distinctively different from ionic, metallic, and covalent solids. We have thus suggested to call these solids with a unique property portfolio “metavalent” to express that these electron‐deficient solids are located between metals and covalent solids, yet have properties that are distinctively different from both. It is possibly most surprising how well these solids can be distinguished from ordinary covalent solids.^[^ ^31^ ^]^ This is the second reason why we chose the word “metavalent,” since this implies that these materials are beyond (Greek “meta”) covalent solids.

The differences between GeTe and the molecular crystals discussed here (XeF_2_ and B_2_H_6_) extend beyond the differences in band structure and the presence (for GeTe) or the absence (for XeF_2_ and B_2_H_6_) of a Peierls distortion. There are also large differences in the optical dielectric constant ε_∞_. For both quantities, GeTe shows characteristically high values, while significantly lower values are found for XeF_2_ and B_2_H_6_.

In this section, we have shown that the properties of electron‐rich molecular crystals such as XeF_2_ and electron‐deficient molecular crystals like B_2_H_6_ differ significantly from electron‐deficient solids like GeTe or PbTe. These chalcogenides are characterized by an infinite chain of overlapping orbitals, where adjacent atoms share ≈1 electron. This electronic configuration frequently leads to electronic instabilities (Peierl distortion). Indeed, many crystalline phase change materials reveal such Peierls distortions which can be interpreted as a competition between electron‐localization and electron‐delocalization. This explains why metavalent solids are located between metals (characterized by electron delocalization) and covalent solids (representative for electron (pair) localization) but possess a distinctively different property portfolio. Interestingly, distinct property changes have been observed upon crossing the border between covalent and metavalent bonding, in line with the claim that metavalent solids form a distinct class.^[^ ^31b^ ^]^ This view has been further corroborated by Arora et al.,^[^ ^29^ ^]^ who employed DFT calculations to show that indeed well‐defined borders exist separating regions of ionic, covalent, metallic, and metavalent bonding.

## 4 Summary

In summary, quantum chemistry has reached a level of maturity that enables the quantification of bonding in solids with high precision. This is demonstrated here, where it is shown in Figure 1 that density‐based and orbital‐based approaches provide a coherent view of bonding. Two fundamental parameters, the number of electrons transferred and shared between adjacent atoms characterize the bonding in solids. The resulting map distinguishes regions of ionic, covalent, and metallic bonding. Between these regions an area is located where electron‐deficient solids like GeTe or PbTe are found, whereas hypervalent solids such as XeF_2_ are located in a distinctively different region of the map. The electron‐deficient solids mentioned above are characterized by a unique combination of properties including high Born effective charges ( Z* =  4 − 6) as a measure of their chemical bond polarizability, moderate electrical conductivities (σ  = 10^1^  − 10^4^ *S* *cm* − 1), characteristic for the intermediate level of electron delocalization, high dielectric constants (ε_∞_ > 15), indicative of the electronic polarizability as well as high Grüneisen parameters (γ_TO_ > 3), characteristic for their pronounced anharmonicity. This property portfolio is distinctively different from metallic, ionic, and covalent bonding, necessitating a new name. We believe that metavalent bonding is a good name for this bond type, while we have shown that the term “hypervalent (electron‐rich 3c–4e)” bonding instead is inadequate here. Metavalent solids are characterized by the competition between electron localization and delocalization. Their pronounced dependence upon external parameters such as temperature and pressure identifies them as quantum materials par excellence.

We note in passing that recently also the name electron‐deficient multi‐center bonding has been suggested to label these solids. While the word electron‐deficient bonding seems adequate, this is less obvious for the label “multi‐center” bonding. After all, metallic bonding would also qualify as electron‐deficient multicenter bonding. Hence this terminology is not suited to describe and understand the border expected between metavalent and metallic bonding. This border could help obtain new insights into the transition between the insulating and the metallic state, a transition that has fascinated generations of physicists including Phil Anderson^[^ ^32^ ^]^ and Nevil Mott.^[^ ^33^ ^]^ The border between the metavalent and metallic regions might offer another pathway from insulators to metals,^[^ ^34^ ^]^ that is enriched by the topology of the electronic structure.

Achieving such an understanding seems to be the real promise of the maps shown in Figure 1. So far, bonding concepts in solids have been the focus of quantum chemistry, while discussing band structures has found a home in solid‐state physics. We expect strong links between bonding and band structures to emerge in the near future for advanced functional materials such as thermoelectrics, photovoltaics, and phase change materials facilitated by the concepts behind Figure 1. In particular, we expect that systematic changes in the quantum–chemical bonding descriptors lead to systematic changes in band structures and resulting material properties, providing a novel avenue for materials design. At the same time, systematic change of material properties as a function of the chemical bonding descriptors (ES and ET) can help to unravel the nature of chemical bonding, reconciling the concepts of quantum chemistry (chemical bonding) and solids state physics (band structures) in their mission to design functional materials.

## Conflict of Interest

The authors declare no conflict of interest.

## Supporting information

Supporting Information
