# Supplementary material for: Metavalent or Hypervalent Bonding: Is There a Chance for Reconciliation?
Source: Adv Sci (Weinh). 2023 Dec 7;11(6):2308578. doi: 10.1002/advs.202308578 (PMC10853697; doi:10.1002/advs.202308578)
Supplement: Supplementary file 1 — Supporting Information [file ADVS-11-2308578-s001.pdf]

ADVANCED SCIENCE
Open Access

Supporting Information

for *Adv. Sci.*, DOI 10.1002/advs.202308578

Metavalent or Hypervalent Bonding: Is There a Chance for Reconciliation?

Matthias Wuttig, Carl-Friedrich Schön, Dasol Kim, Pavlo Golub, Carlo Gatti, Jean-Yves Raty, Bart J. Kooi, Ángel Martín Pendás, Raagya Arora and Umesh Waghmare*

Supplemental Information:

Metavalent or Hypervalent Bonding: Is there a chance for reconciliation?

Matthias Wuttig[1,2,3], Carl-Friedrich Schön[1], Dasol Kim[1], Pavlo Golub[4], Carlo Gatti[5], Jean-Yves Raty[6], Bart Kooi[7], A. Martín Pendás[8], Raagya Arora[9], Umesh Waghmare[9]

1) I. Institute of Physics, Physics of Novel Materials, RWTH Aachen University, 52056 Aachen, Germany
2) Jülich-Aachen Research Alliance (JARA FIT and JARA HPC), RWTH Aachen University, 52056 Aachen, Germany
3) PGI 10 (Green IT), Forschungszentrum Jülich GmbH, 52428 Jülich, Germany
4) J. Heyrovský Institute of Physical Chemistry, Department of Theoretical Chemistry, Dolejškova 2155/3, 182 23 Prague 8, Czech Republic
5) CNR-SCITEC, Istituto di Scienze e Tecnologie Chimiche "Giulio Natta", sezione di via Golgi, via Golgi 19, 20133 Milano, Italy
6) CESAM B5, Université de Liège, B4000 Sart-Tilman, Belgium
7) Zernike Institute for Advanced Materials, University of Groningen, Nijenborgh 4, Groningen 9747 AG, the Netherlands
8) Dept. de Química Física y Analítica. Julián Clavería 8. 33006 Oviedo, Spain
9) Theoretical Sciences Unit, School of Advanced Materials, JNCASR, Jakkur, Bangalore 560 064, India

Keywords: *quantum chemical bonding descriptors, hypervalent bonding, metavalent bonding, material design, material maps*

Table of Contents:

I. Quantum Chemical Analysis of Bonding in XeF_2 (including DAFH table, $5d_{z^2}$ -orbital contribution of Xe) in comparison with GeTe.

II. Quantum Chemical Analysis of Bonding in ClF_3 and SF_4

III. Contribution of s- states to bonding in GeTe and related chalcogenides as well as pnictogens

I. Quantum Chemical Analysis of Bonding in XeF_2 (including DAFH table, $5d_{z^2}$ - orbital contribution of Xe) in comparison with GeTe.

XeF_2 (Molecular crystal)

Xe

DAFH Orbitals	Occupation, e	Localization within native basin	Localization within bond partner basin	DI contribution (Xe-F)
$5p_x$	0.75	37.5%	30.85% (2x)	0.46
5p (2x)	1.91	95.7%	1.09% (2x)	0.04
5s	1.95	97.3%	1.21% (2x)	0.05

F(1)

DAFH Orbitals	Occupation, e	Localization within native basin	Localization within bond partner basin	DI contribution (Xe-F)
$2p_x$	1.56	77.93%	17.16%	0.535
2p (2x)	1.94	96.83%	1.79%	0.069
2s	1.99	99.55%	0.24%	0.009

F(2)

DAFH Orbitals	Occupation, e	Localization within native basin	Localization within bond partner basin	DI contribution (X-F)
$2p_x$	1.56	77.93%	17.16%	0.535
2p (2x)	1.94	96.83%	1.79%	0.069
2s	1.99	99.55%	0.24%	0.009

Table 1: Localization within bond partner basin calculated from a DAFH orbital analysis for XeF_2. The data in the table describe the contribution of each single DAFH orbital. For the p- orbitals there are significant contributions to two opposite neighbors with identical localization. The contribution due to s - p- orbital overlap is negligible. In addition, a significant orbital contribution exists for the Xe atom, which is involved in bonding. For the XeF_2 molecule, this adds up to a DI of 0.11.

GeTe:

Ge

DAFH Orbitals	Occupation, e	Localization within native basin	Localization within bond partner basin	DI contribution
4p (3x)	0.51	27.5%	27.32% (2x)	0.28 (Ge-Te)
4s	1.74	87.7%	1.7% (6x)	0.06
3d (5×)	2	>99.9%	-	-

Te

DAFH Orbitals	Occupation, e	Localization within native basin	Localization within bond partner basin	DI contribution
5p (3x)	1.31	66%	11.4% (2x)	0.299 (Te-Ge)
5s	1.88	94.3%	0.8% (6x)	0.029 (Te-Ge)

Table 2: Localization within bond partner basin calculated from a DAFH orbital analysis for cubic GeTe using the atomic arrangement of the materials project data file (mp_2612) and subsequent structural relaxation. The data in the table describe the contribution of each single DAFH orbital. Bonding is governed by the p-orbital overlap.

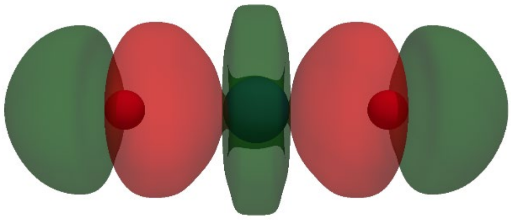

Figure S1: Visualization of the DAFH orbital corresponding to $5d_{z^2}$ orbital of Xe. The isosurfaces have been set to 0.001 (red) and -0.001 (green) respectively.

II. Quantum Chemical Analysis of Bonding in ClF_3 and SF_4

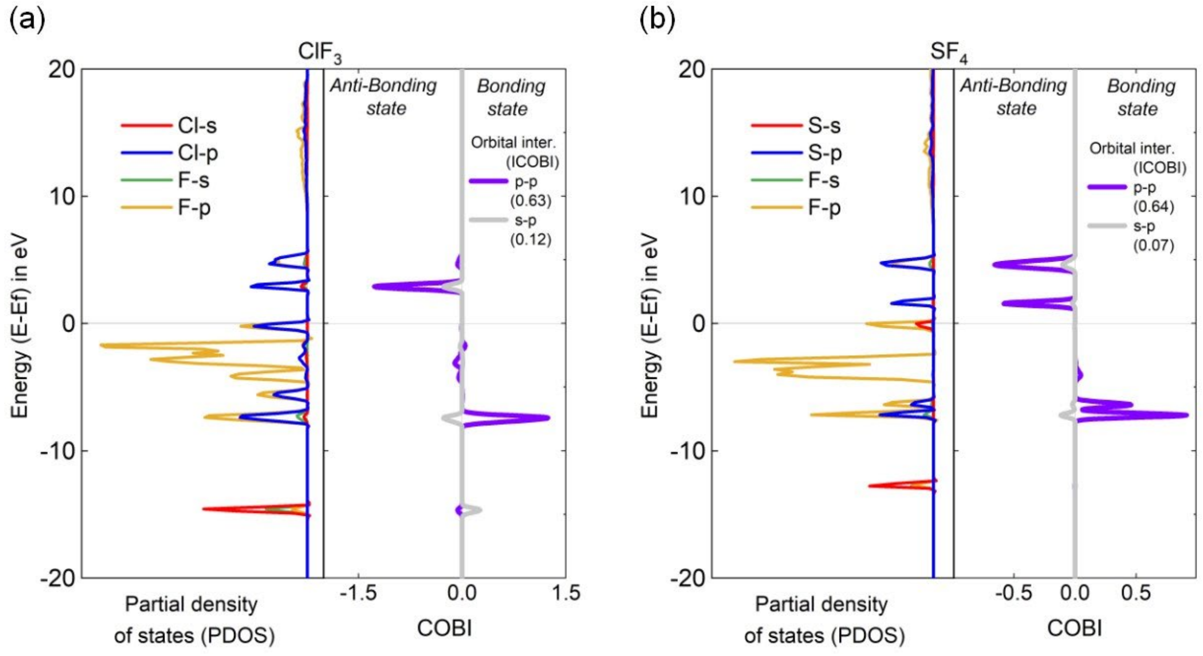

Figure S2: Visualization of bonding analysis for two other hypervalent molecular crystals. Both solids have a bonder order with clearly exceeds 0.5, i.e. clearly have a larger ES value than 1.

III. Contribution of s-states to bonding in GeTe, related chalcogenides and pnictogens

Frequently, the belief has been expressed that the s-electrons in monochalcogenides play a significant role. Sometimes this potential contribution is described as a lone pair contribution. This idea at first seems to be supported by the COBI data shown in figure S3, where an antibonding feature, due to s- p orbital overlap right below E_F is shown for GeTe. As shown in figure S4, such an antibonding state is not found for Si, but can be identified in many mono-chalcogenides. Hence, one can ponder if this antibonding feature is crucial for these chalcogenides, i.e. if it determines their structure, bonding and properties. To confirm or refute this suspicion, it is necessary to understand the origin of the antibonding contribution. Figure S3 shows that this antibonding contribution comes from the orbital overlap between group IV s-electrons with group VI p-electrons. This orbital overlap is found for all monochalcogenides independent of the elements employ (for Ge, Sn and Pb formal cations and S, Se and Te formal anions. The overlap also exists independent of crystal structure, i.e. cubic, rhombohedral and orthorhombic systems. This s – p -orbital overlap is unavoidable for monochalcogenides (and also exists for Sb and Bi), since it develops automatically since there is always some limited overlap between these s- and p- orbitals. This has been convincingly discussed in a paper by Papoian and Hoffmann [1]. They argue that the size of this orbital overlap is governed by two factors [2], the energy difference and the spatial overlap of these two orbitals. Concerning the energy difference, the impact on the orbital overlap is strong, if the energy difference is small. As shown in the DoS, for the monochalcogenides this energy difference is typically modest. Hence, it is not dominant for heavy chalcogenides. The second important factor is the spatial overlap between the two orbitals. In heavy elements, the s-orbital is usually significantly smaller that the corresponding p-orbital, a relativistic effect. Hence, the overlap between the Ge s- and Te p- state is quite small. Since the spatial overlap is even more important than the energy difference of the orbitals involved, the s-p orbital overlap in the higher chalcogenides is not governing the bonding and properties of these materials [3]. In particular, this contribution does not drive the Peierls distortion, as also supported by figure S3. This conclusion is perfectly in line with qualitative arguments presented already two decades ago by Papoian and Hoffmann [1]. Now we can confirm and quantify this view by accurate quantum-chemical calculations of bonding in solids.

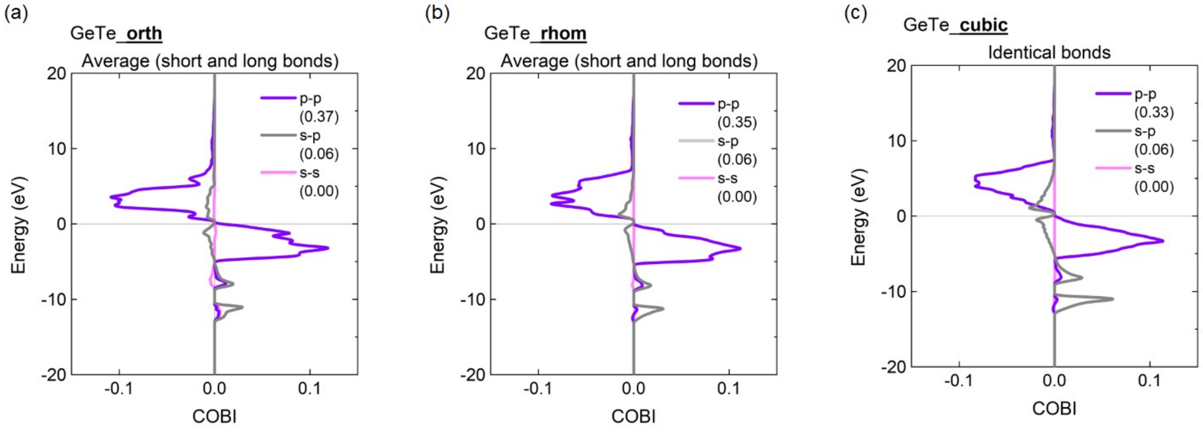

Figure S3: COBI bonding analysis for different levels of Peierls distortion in GeTe, from the orthorhombic phase (large distortion), to the rhombohedral phase (moderate distortion) to the cubic phase (no Peierls distortion). In all cases, there is a small contribution of s- p orbital overlap, as quantified by the ICOBI, which does not change upon increasing Peierls distortion. Instead, there is a change for the p – p orbital overlap, which also governs the band gap opening.

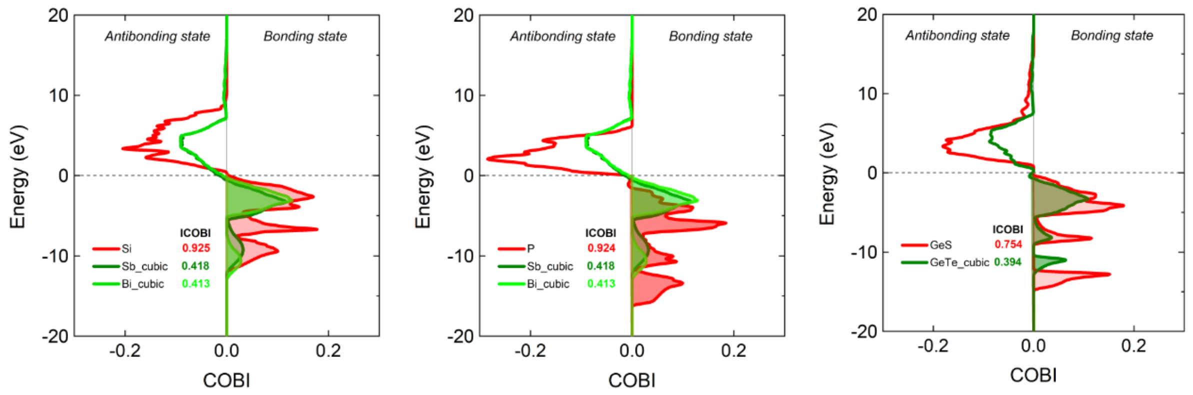

Figure S4: COBI bonding analysis for different solids, including Si, (simple) cubic Sb and Bi, as well as GeSe (orthorhombic) and GeTe(cubic). Only in Si, no antibonding state is occupied in the vicinity of the Fermi energy E_F. All other solids show a small antibonding state close to the Fermi level, due to s- p orbital overlap, as already discussed previously [1]. Significant changes in p – orbital overlap are observed for the different p- bonded solids (Sb, Bi, GeTe, GeSe). These changes are closely related to the Peierls distortion and modify the properties of these solids.

References:

[1] G. A. Papoian, R. Hoffmann, *Angew. Chem.-Int. Edit. Engl.* **2000**, 39, 2408.
[2] A. Imamura, Mol. Phys. **1968**, 15, 225.
[3] C. Froese Fischer, At. Data **1972**, 4, 301
